# Chloroplast DNA sequence of the green alga *Oedogonium cardiacum *(Chlorophyceae): Unique genome architecture, derived characters shared with the Chaetophorales and novel genes acquired through horizontal transfer

**DOI:** 10.1186/1471-2164-9-290

**Published:** 2008-06-16

**Authors:** Jean-Simon Brouard, Christian Otis, Claude Lemieux, Monique Turmel

**Affiliations:** 1 Département de biochimie et de microbiologie, Université Laval, Québec, QC G1V 0A6, Canada

## Abstract

**Background:**

To gain insight into the branching order of the five main lineages currently recognized in the green algal class Chlorophyceae and to expand our understanding of chloroplast genome evolution, we have undertaken the sequencing of chloroplast DNA (cpDNA) from representative taxa. The complete cpDNA sequences previously reported for *Chlamydomonas *(Chlamydomonadales), *Scenedesmus *(Sphaeropleales), and *Stigeoclonium *(Chaetophorales) revealed tremendous variability in their architecture, the retention of only few ancestral gene clusters, and derived clusters shared by *Chlamydomonas *and *Scenedesmus*. Unexpectedly, our recent phylogenies inferred from these cpDNAs and the partial sequences of three other chlorophycean cpDNAs disclosed two major clades, one uniting the Chlamydomonadales and Sphaeropleales (CS clade) and the other uniting the Oedogoniales, Chaetophorales and Chaetopeltidales (OCC clade). Although molecular signatures provided strong support for this dichotomy and for the branching of the Oedogoniales as the earliest-diverging lineage of the OCC clade, more data are required to validate these phylogenies. We describe here the complete cpDNA sequence of *Oedogonium cardiacum *(Oedogoniales).

**Results:**

Like its three chlorophycean homologues, the 196,547-bp *Oedogonium *chloroplast genome displays a distinctive architecture. This genome is one of the most compact among photosynthetic chlorophytes. It has an atypical quadripartite structure, is intron-rich (17 group I and 4 group II introns), and displays 99 different conserved genes and four long open reading frames (ORFs), three of which are clustered in the spacious inverted repeat of 35,493 bp. Intriguingly, two of these ORFs (*int *and *dpoB*) revealed high similarities to genes not usually found in cpDNA. At the gene content and gene order levels, the *Oedogonium *genome most closely resembles its *Stigeoclonium *counterpart. Characters shared by these chlorophyceans but missing in members of the CS clade include the retention of *psaM*, *rpl32 *and *trnL*(caa), the loss of *petA*, the disruption of three ancestral clusters and the presence of five derived gene clusters.

**Conclusion:**

The *Oedogonium *chloroplast genome disclosed additional characters that bolster the evidence for a close alliance between the Oedogoniales and Chaetophorales. Our unprecedented finding of *int *and *dpoB *in this cpDNA provides a clear example that novel genes were acquired by the chloroplast genome through horizontal transfers, possibly from a mitochondrial genome donor.

## Background

The Chlorophyceae (sensu Mattox and Stewart) is a morphologically diverse class of green algae, which together with three other green algal classes (Prasinophyceae, Trebouxiophyceae and Ulvophyceae) form the Chlorophyta [1]. The Chlorophyceae and Ulvophyceae are presumed to share a sister-relationship, with the Trebouxiophyceae being sister to the Chlorophyceae + Ulvophyceae clade and the Prasinophyceae representing the earliest offshoot of the Chlorophyta [2,3]. Members of the Chlorophyceae display the most variability in terms of the arrangement of the flagellar apparatus. The flagellar basal bodies of motile cells (vegetative cells, zoospores or gametes) are generally directly opposed (DO) or displaced in a clockwise (CW) direction [4]; however, those of the chlorophyceans assigned to the order Oedogoniales display a unique arrangement characterized by an anterior ring of flagella [5]. Phylogenetic analyses of 18S rDNA data and combined 18S and 26S rDNA data from a broad range of chlorophyceans uncovered five major monophyletic groups in the Chlorophyceae: the Chlamydomonadales, Sphaeropleales, Oedogoniales, Chaetophorales, and Chaetopeltidales. The interrelationships of these chlorophycean lineages, however, could not be unraveled. Although most internal nodes in the trees inferred from the 18S and 26S rDNA data received poor support, the five lineages were most often recovered as a grade, with the sister-relationship observed for the Chlamydomonadales and Sphaeropleales being the best supported [6-9].

Comparative analyses of chloroplast genomes have proven very useful to clarify the evolutionary relationships among the main groups of green algae and land plants [10-18]. We adopted this strategy to gain insight into the branching order of chlorophycean lineages and also to better understand the forces accounting for the very fluid structure of the chloroplast genome in the Chlorophyceae. We recently described the complete chloroplast genomes of *Scenedesmus obliquus *(Sphaeropleales) [19] and *Stigeoclonium helveticum *(Chaetophorales) [20] and compared them to their homologue in *Chlamydomonas reinhardtii *(Chlamydomonadales) [21]. All three genomes have retained only a few of the ancestral characters observed in their counterparts from other classes. Their reduced gene repertoires, which comprise 94 to 97 genes, lack six of the protein-coding genes (*accD*, *chlI*, *minD*, *psaI*, *rpl19*, and *ycf20*) identified in *Pseudendoclonium akinetum *and *Oltmannsiellopsis viridis*, the two members of the Ulvophyceae whose chloroplast genomes have been examined so far [12,22]. Some genes show unique alterations at the structural level. In all three chlorophyceans, the *rpoB *gene is split into two distinct open reading frames (ORFs) and the *clpP *and *rps3 *genes have unusually large coding regions. Moreover, the coding region of *rps4 *in *Stigeoclonium *displays a prominent insertion that is apparently devoid of any intron or intein. Both the *Chlamydomonas *and *Scenedesmus *genomes possess a large inverted repeat (IR) encoding the rRNA operons; however, the gene contents of their single copy (SC) regions are entirely different and do not conform to the ancestral quadripartite structure observed in the prasinophycean *Nephroselmis olivacea *[23] and in most streptophyte green algae [11,16,24,25] and land plants [26,27]. Despite these differences, they share 11 gene clusters that have not been previously observed in other green algae. The IR-lacking chloroplast genome of *Stigeoclonium *is extremely rearranged relative to its chlorophycean counterparts and exhibits a number of distinctive traits, including four putatively *trans*-spliced group II introns inserted in *petD*, *psaC *and *rbcL *as well as a remarkably strong bias in gene coding regions and base composition of the two DNA strands. Both the strand biases in coding regions and G + C composition were found to be typical of those observed in prokaryotic genomes that replicate bidirectionally from a single origin.

More recently, we used the sequence data of the three abovementioned chlorophycean chloroplast genomes together with those of the partly sequenced chloroplast DNAs (cpDNAs) of *Chlamydomonas moewusii *(Chlamydomonadales), *Oedogonium cardiacum *(Oedogoniales) and *Floydiella terrestris *(Chaetopeltidales) to reconstruct trees from nucleotide and amino acid data sets derived from more than 40 protein-coding genes [15]. All best trees identified two robustly supported lineages within the Chlorophyceae: a clade uniting the Chlamydomonadales and Sphaeropleales (CS clade) and a clade uniting the Oedogoniales, Chaetophorales, and Chaetopeltidales (OCC clade). This dichotomy was independently supported by molecular signatures in chloroplast genes such as insertions/deletions and the distribution of *trans*-spliced group II introns [15]. Within the OCC clade, the sister relationship observed for the Chaetophorales and Chaetopeltidales was strengthened by the presence of *trans*-spliced group II introns at two identical positions in the *Stigeoclonium *and *Floydiella rbcL *genes. Nevertheless, the possibility that the Chaetophorales are sister to the Oedogoniales + Chaetopeltidales clade could not be rejected by statistical tests. Character state reconstruction of basal body orientation using the best tree as phylogenetic framework predicted that the last common ancestor of all chlorophycean green algae featured quadriflagellate motile cells with the DO + DO orientation and that changes to the CW condition occurred convergently in the CS and OCC clades [15].

We present here the complete chloroplast genome sequence of *Oedogonium *(Oedogoniales) and report additional genomic characters bolstering the evidence for a close alliance between the Oedogoniales and Chaetophorales. Like its *Stigeoclonium*, *Chlamydomonas *and *Scenedesmus *homologues, the *Oedogonium *genome displays a distinctive architecture. Although this IR-containing chloroplast genome is highly shuffled in gene order relative to the other completely sequenced chlorophycean genomes, it shares unique gene clusters with the *Stigeoclonium *genome as well as the retention and loss of specific genes. To our surprise, we identified three genes that were acquired by horizontal gene transfer in a 10-kb region of the exceptionally large IR.

## Results

### Overall structure and gene arrangement

**Figure 1:**
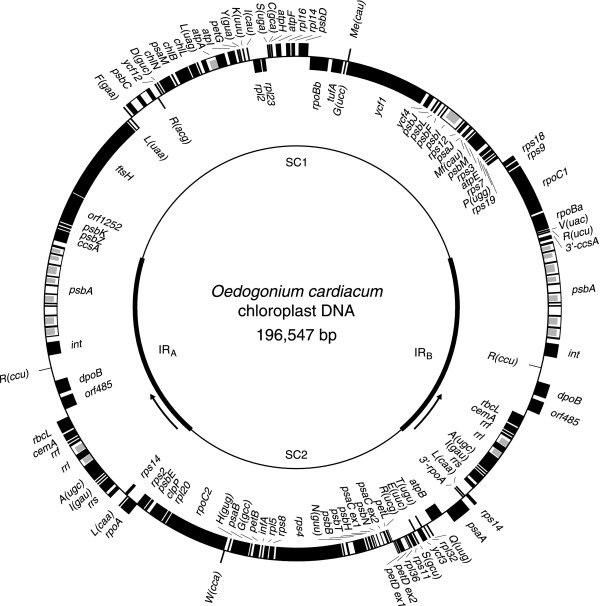
**Gene map of *Oedogonium *cpDNA**. Thick lines on the inner circle represent the two copies of the IR (IR_A _and IR_B_), with the arrows indicating the transcription direction of the IR-encoded rRNA operon. Genes (filled boxes) on the outside of the outer circle are transcribed in a clockwise direction. Introns are represented by open boxes and intron ORFs are denoted by narrow, filled boxes. The *rpoB *gene consists of two separate ORFs (*rpoBa *and *rpoBb*) that are not associated with sequences typical of group I or group II introns. tRNA genes are indicated by the one-letter amino acid code followed by the anticodon in parentheses (Me, elongator methionine; Mf, initiator methionine). Only one of the two isomeric forms of the genome is shown here; these isomers differ with respect to the relative orientation of the SC regions.

The *Oedogonium *chloroplast genome assembles as a circle of 196,547 bp with an A+T content of 70.5% (Fig. 1). As shown in Table 1, these values fall within the range of sizes and base compositions observed for the three previously sequenced chlorophycean cpDNAs (Table 1). The *Oedogonium *genome displays two identical copies of a 35,492-bp IR that are separated from one another by SC regions of 80,363 and 45,200 bp, designated here SC1 and SC2, respectively. Note that the latter regions are not designated as large and small SC (LSC and SSC) regions as is the case for *Nephroselmis *and streptophyte cpDNAs because their gene contents deviate considerably from the ancestral partitioning pattern featured by these genomes [23,28]. In addition, the quadripartite structure of the *Oedogonium *genome differs greatly from those of its *Chlamydomonas *and *Scenedesmus *homologues with respect to the gene contents of the IR and the SC regions and also the relative sizes of these three main genomic regions (Table 1). In *Oedogonium*, the IR is significantly larger than most of its green algal and land plants counterparts and the vastly unequal sizes of the SC regions contrast with the similar sizes of the same regions in *Chlamydomonas *and *Scenedesmus *cpDNAs.

**Table 1:** General features of *Oedogonium *and other chlorophycean cpDNAs

**Feature**	***Oedogonium***	***Stigeoclonium***	***Scenedesmus***	***Chlamydomonas***
Size (bp)				
Total	196,547	223,902	161,452	203,827
IR	35,492	-^a^	12,022	22,211
SC1^b^	80,363	-^a^	72,440	81,307
SC2^c^	45,200	-^a^	64,968	78,088
A+T (%)	70.5	71.1	73.1	65.5
Sidedness index	0.74	0.95	0.88	0.87
Conserved genes (no.) ^d^	99	97	96	94
Introns				
Fraction of genome (%)	17.9	7.9	8.6	6.8
Group I (no.)	17	16	7	5
Group II (no.)	4	5	2	2
Intergenic sequences ^e^				
Fraction of genome (%)	22.6	46.7	34.3	49.2
Average size (bp)	370	1026	517	937
Short repeated sequences				
Fraction of genome (%) ^f^	1.3	17.8	3.0	15.8

^a ^Because *Stigeoclonium *cpDNA lacks an IR, only the total size of this genome is given.

^b ^SC region with the larger size.

^c ^SC region with the smaller size.

^d ^Conserved genes refer to free-standing coding sequences usually present in chloroplast genomes. Genes present in the IR were counted only once.

^e ^The *Oedogonium orf485 *and the ORFs encoding the maturase domain of reverse transcriptase in *Oedogonium *and *Scenedesmus *(*orf1252 *and *orf932*, respectively) were considered as gene sequences.

^f ^Non-overlapping repeated elements were identified as described in the Methods.

The 99 conserved genes and four free-standing ORFs of more than 100 codons found in *Oedogonium *cpDNA are densely packed (Fig. 1). The intergenic spacers account for 22.6% of the total genome sequence and vary from 22 to 1721 bp, for an average size of 370 bp. This is the lowest proportion and smallest average size of intergenic spacers observed thus far for the chloroplast genome of a photosynthetic chlorophyte belonging to the Ulvophyceae, Trebouxiophyceae or Chlorophyceae (UTC) (Table 1 and [29]).

The *Oedogonium *IR contains six conserved genes and three ORFs in addition to the rRNA-encoding operon, whereas the SC1 and SC2 regions encode 54 and 34 genes, respectively. The SC1 and SC2 boundaries of the IR lie in the 3'ends of *ccsA *and *rpoA*, respectively. Interestingly, the 3'end of *trnR*(ucu) encompasses the SC1 boundary of the IR; the last two bp of this gene coding region maps to the IR and are thus shared with *ccsA*. These genes were considered as part of the IR in the breakdown of genes provided above. The rRNA operon lies at the proximity of the SC2 region and is transcribed toward to the largest SC region (SC1). In contrast, the rRNA operon is always transcribed toward the smallest SC region in cpDNAs that have retained the ancestral quadripartite structure. In this context, it is worth mentioning that the chloroplast genome of the ulvophycean *Pseudendoclonium*, which carries an atypical quadripartite structure, resembles its *Oedogonium *homologue in exhibiting an IR whose rRNA operon is transcribed toward the largest SC region [12]. Considering that the latter region in *Pseudendoclonium *is clearly equivalent to the LSC region found in genomes having an ancestral quadripartite structure, it was speculated that a change in the transcription direction of the rRNA operon might have occurred in conjunction with the exchange of a few genes between the two SC regions. Subsequent analysis of an ulvophycean belonging to a separate early-branching lineage (*Oltmannsiellopis*) suggested that these genomic events might have occurred before or soon after the emergence of the Ulvophyceae [22]. Unlike their ulvophycean homologues, the *Oedogonium *SC regions display no remnant of an ancestral gene partitioning pattern, nor do they bear any obvious similarities in gene content to the SC regions in either *Chlamydomonas *or *Scenedesmus *cpDNA.

A strong bias in the distribution of genes between the two DNA strands has been noted in the *Stigeoclonium *chloroplast genome [20] and, to a lesser extent, in its *Scenedesmus *counterpart [19]. The propensity of contiguous genes to be encoded on the same strand can be estimated using the sidedness index [30]. Even though several gene clusters are found on the same strand in *Oedogonium *cpDNA (Fig. 1), the calculated value of 0.74 for the sidedness index indicates that this is the chlorophycean genome where this tendency is the least pronounced (Table 1).

### Conserved genes and ORFs

The 99 conserved genes of *Oedogonium *represent the largest gene repertoire among the four chlorophycean cpDNAs completely sequenced to date (Table 1). A common set of 93 genes, including a fragmented *rpoB*, are shared by these genomes (Table 2). The collection of conserved genes displayed by *Oedogonium *closely resembles that of *Stigeoclonium*, the only differences being the presence of three extra genes [*infA*, *trnR*(ccu) and *trnR*(ucg)] and the absence of *trnS*(gga) in the former alga (Table 2). Compared to the representatives of the CS clade, both *Oedogonium *and *Stigeoclonium *feature three additional genes [*psaM*, *rpl32 *and *trnL*(caa)] and lack *petA*. These members of the OCC clade also differ from those of the CS clade in exhibiting insertions of more 2,500 codons (2,562 codons in *Oedogonium *and 2,682 codons in *Stigeoclonium*) immediately before the 3'conserved region of *rps4*. The sequences of these insertions are extremely divergent, thereby precluding us from decrypting their nature. Intriguingly, the corresponding 3' regions of the *Chlamydomonas *and *Scenedesmus rps4 *genes are missing not only a large insertion but also the highly conserved sequence corresponding to the last 40 codons [15].

**Table 2:** Differences between the repertoires of conserved genes in *Oedogonium *and other chlorophycean cpDNAs

**Gene^a^**	***Oedogonium***	***Stigeoclonium***	***Scenedesmus***	***Chlamydomonas***
*infA*	+	-	+	-
*petA*	-	-	+	+
*psaM*	+	+	-	-
*rpl12*	-	-	+	-
*rpl32*	+	+	-	-
*trnL*(caa)	+	+	-	-
*trnR*(ccu)	+	-	-	-
*trnR*(ucg)	+	-	-	-
*trnS*(gga)	-	+	-	-

^a ^Only the genes that are missing in one or more genomes are indicated. Plus and minus signs denote the presence and absence of genes, respectively. A total of 93 genes are shared by all compared cpDNAs: *atpA, B, E, F, H, I, ccsA, cemA, chlB, L, N, clpP, ftsH, petB, D, G, L, psaA, B, C, J, psbA, B, C, D, E, F, H, I, J, K, L, M, N, T, Z, rbcL, rpl2, 5, 14, 16, 20, 23, 36, rpoA, B, C1, C2, rps2, 3, 4, 7, 8, 9, 11, 12, 14, 18, 19, rrf, rrl, rrs, tufA, ycf1, 3, 4, 12, trnA*(ugc), *C*(gca), *D*(guc), *E*(uuc), *F*(gaa), *G*(gcc), *G*(ucc), *H*(gug), *I*(cau), *I*(gau), *K*(uuu), *L*(uaa), *L*(uag), *Me*(cau), *Mf*(cau), *N*(guu), *P*(ugg), *Q*(uug), *R*(acg), *R*(ucu), *S*(gcu), *S*(uga), *T*(ugu), *V*(uac), *W*(cca) and *Y*(gua).

As mentioned above, four free-standing ORFs in *Oedogonium *cpDNA showed no sequence similarity with any genes usually present in chloroplast genomes. Three of these ORFs (*orf485*, *orf512 *and *orf538*) are clustered in the central region of the IR. The contiguous *orf485 *and *orf538 *(*dpoB *in Fig. 1) reside on the same strand as *orf512 *(*int *in Fig. 1) but are separated from this ORF by *trnR*(ccu), a gene located on the opposite strand. The fourth ORF (*orf1252*) is located within the SC1 region between *ftsH *and *psbK*. For all four ORFs, except *orf485*, our Blast searches identified similarities with genes encoding proteins acting on DNA or RNA.

We found that the *orf512 *encodes a protein belonging to the family of tyrosine recombinases. The functions of these site-specific recombinases include the integrative and excisive recombination of viral and plasmid DNA into and out of the host genome, conjugative transposition, resolution of catenated DNA circles, regulation of plasmid copy number, and DNA inversions controlling the expression of cell surface proteins or DNA replication [31]. The C-terminal region of the *orf512 *product displays the essential tyrosine and the five other active site residues that are diagnostic of this protein family [31,32]. BlastP searches identified the catalytic domain found in the family of phage integrases (pfamH00589) as being the most highly conserved in sequence with the C-terminal region of the *orf512*-encoded protein. The members of the tyrosine recombinase family that proved the most similar to the *Oedogonium orf512 *product are the proteins of unknown function encoded by the mitochondrial genomes of two green algae, the charophycean *Chaetosphaeridium globosum *[25] (*E*-value threshold of 7 × 10^-8^) and the trebouxiophycean *Prototheca wickerhamii *[33] (*E*-value threshold of 3 × 10^-6^).

The *orf538 *encodes a member of the B family of DNA-directed DNA polymerases. These polymerases were named for their homology with the product of the *polB *gene encoding *Escherichia coli *polymerase II. Members of this family are extensive in number and variety, occurring in prokaryotes, eukaryotes and viruses [34]. [35]. The DNA polymerase encoded by a linear mitochondrial plasmid in the fungus *Neurospora intermedia *(the kalilo element; [36]) revealed the highest level of sequence similarity (*E*-value threshold of 7 × 10^-32^) with the *orf538 *product.

BlastP searches with the *orf485*-encoded protein disclosed limited similarity (*E*-value threshold of 7 × 10^-5^) with the conserved domain of the septation ring formation regulator ErzA (pfam06160), a protein that modulates the frequency and position of FtsZ ring formation during the bacterial cell cycle. Because the detected similarity is marginal and restricted to a portion of the ErzA domain, the *orf485 *product is unlikely to function as an ErzA protein.

Finally, a short C-terminal region of the *orf1252 *product revealed sequence similarity with the maturase domain of reverse transcriptases encoded by bacterial group II introns and also with the maturase domain of the protein predicted from the free-standing *orf932 *in *Scenedesmus *cpDNA [19]. Although they carry the maturase domain, both the *Oedogonium orf1252 *and *Scenedesmus orf932 *products lack the reverse transcriptase domain.

Each of the four *Oedogonium *ORFs was compared with the set of 68 protein-coding genes to assess their similarity in codon usage (Table 3). We found that the *orf512/int *differs markedly in base composition at the first and third codon positions relative to conserved protein-coding genes; moreover, the codon adaptation index (CAI) [37] calculated for this ORF deviates considerably from the average value obtained for the conserved genes. The *orf1252 *also differs greatly from conserved protein-coding genes with respect to base composition, but its CAI is not significantly different. For the *orf485 *and *orf538/dpoB*, only moderate differences were observed for both the base composition and CAI relative to the conserved protein-coding genes.

**Table 3:** Compared codon usage of the free-standing ORFs and conserved protein-coding genes in *Oedogonium *cpDNA

	**G+C content in codons (%)**			
		
**ORF**	**First position**	**Second position**	**Third position**	**CAI**
*orf485*	38.5	29.8	14.4	0.686
*orf512/int*	48.0	34.3	32.9	0.465
*orf538/dpoB*	33.8	26.4	16.0	0.682
*orf1252*	19.0	17.6	13.3	0.733
Protein-coding genes ^a^	36.5	30.6	14.8	0.723

^a ^Average values obtained from the 68 conserved protein-coding genes.

### Gene order

**Figure 2:**
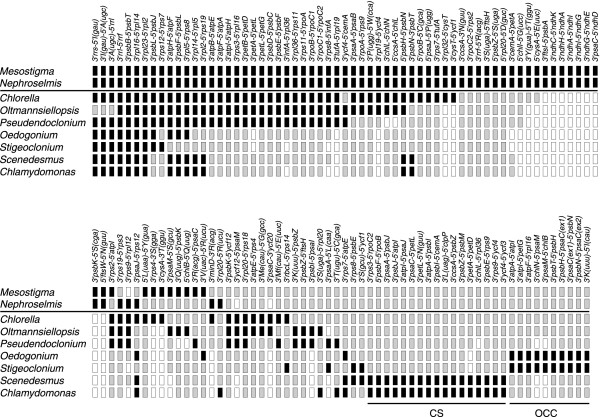
**Conservation of gene pairs in *Oedogonium *and other UTC algal cpDNAs**. For each gene pair, adjoining termini of the genes are indicated. Filled boxes indicate the presence of gene pairs with the same relative polarities in two or more genomes. Grey or open boxes indicate the absence of gene pairs. A grey box indicates that the two genes associated with a gene pair are found in the genome but are unlinked. An open box indicates that one or both genes associated with a gene pair are absent from the genome. Two horizontal lines at the bottom of the figure denote the gene pairs that are shared specifically by *Chlamydomonas *and *Scenedesmus *(CS clade) and by *Oedogonium *and *Stigeoclonium *(OCC clade). The gene pairs shared by the prasinophycean *Nephroselmis *and the streptophyte *Mesostigma viride *were presumably present in the last common ancestor of all green algae. *Chlorella vulgaris *belongs to the Trebouxiophyceae, whereas *Oltmannsiellopsis *and *Pseudendoclonium *are representatives of the Ulvophyceae.

*Stigeoclonium*, *Chlamydomonas *and *Scenedesmus *cpDNAs were previously shown to have retained only a few ancestral gene clusters [20]. As illustrated in Fig. 2, the *Oedogonium *genome has retained about the same set of ancestral clusters. Besides the intact rRNA operon, it displays the *psbF-psbL-psbJ *cluster and six ancestral gene pairs (*atpH-atpF*, *psbB-psbT*, *rpl16-rpl14*, *rpl23-rpl2*, *rpl5-rps8 *and *psaJ-rps12*). The ancestral gene pairs *rpl14-rpl5*, *rpl2-rps19 *as well as the *psbH-psbN-psbT *cluster are missing from both *Oedogonium *and *Stigeoclonium *but are present in both *Scenedesmus *and *Chlamydomonas*.

With regard to the presence of derived gene clusters, the *Oedogonium *chloroplast genome clearly bears more similarity with its *Stigeoclonium *homologue than with the *Chlamydomonas *and *Scenedesmus *cpDNAs. The pentad *psbT-psbH-psaC(ex1)-psbN-psaC(ex2)*, the triads *atpA-atpI-petG *and *chlN-psaM-chlB*, as well as the gene pairs *atpF-rpl16 *and *trnK(uuu)-trnI(cau) *are found in *Oedogonium *and *Stigeoclonium *but are absent from the two members of the CS clade (Fig. 2). Of the 11 derived gene clusters previously reported to be shared by *Chlamydomonas *and *Scenedesmus *but not by *Stigeoclonium*, only one (the pair *rps7-atpE*) was found in *Oedogonium*, suggesting that it arose before the split of the CS and OCC clades. Note here that the order of the coding regions observed for the fragmented *rpoB *gene was not considered in Fig. 2. In this regard, it is interesting to point out that the two *rpoB *gene fragments (*rpoBa *and *rpoBb*) are contiguous in the *Chlamydomonas *and *Scenedesmus *genomes but separated from one another by other genes in the *Oedogonium *and *Stigeoclonium *genomes.

Regardless of their timing of emergence, a total of eight gene clusters are conserved between the *Oedogonium *and *Stigeoclonium *cpDNAs; they encode 26 of the 96 genes shared by these genomes. By comparison, 16 conserved gene clusters in the *Chlamydomonas *and *Scenedesmus *chloroplasts encode 48 of the 94 genes common to these algal genomes.

### Group I Introns

**Figure 3:**
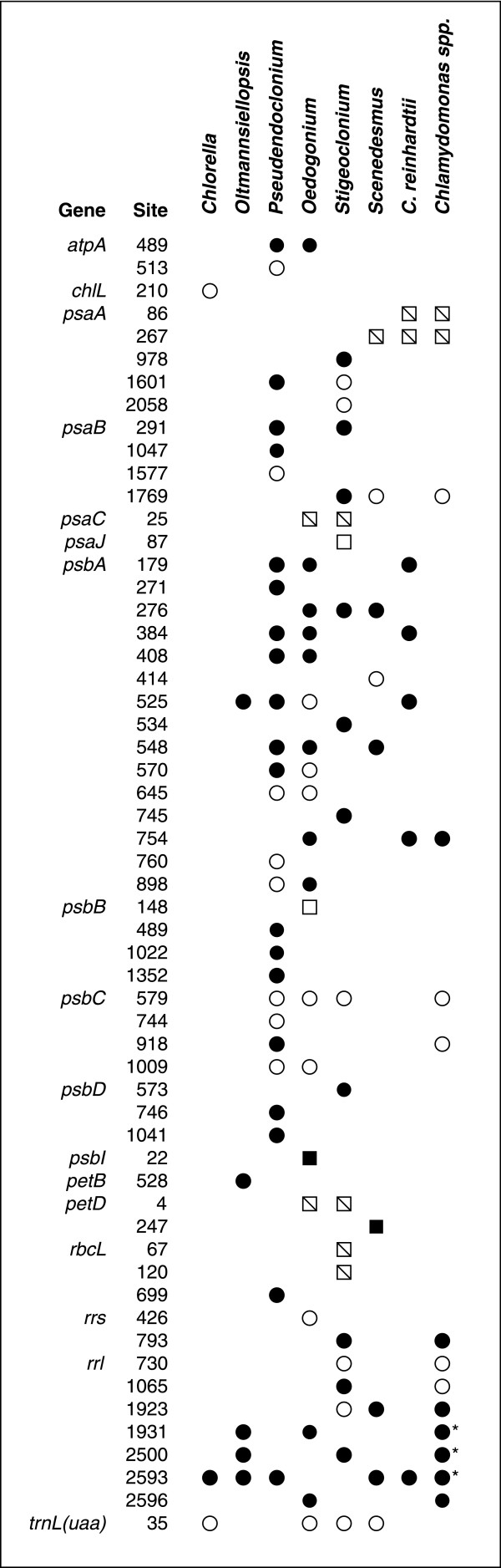
**Distribution of introns in *Oedogonium *and other UTC algal cpDNAs**. Circles denote the presence of group I introns and squares denote the presence of group II introns. Divided squares represent *trans*-spliced group II introns. Open symbols denote the absence of intron ORFs, whereas filled symbols denote their presence. Intron insertion sites are designated as indicated in Table 4. The column at the extreme right indicates the introns of *Chlamydomonas *species other than *C. reinhardtii *that are known to have homologues in completely sequenced UTC algal genomes. References for the latter introns are as follows: *psaB *[70]; *psbA *[71]; *psbC *[70]; *rrs *[72]; and *rrl *[73-76]. An asterisk denotes the absence of the ORF in some *Chlamydomonas *species.

As observed for *Stigeoclonium*, the *Oedogonium *chloroplast genome is rich in introns and most of these genetic elements belong to the group I family (Table 1). The 17 group I introns in the *Oedogonium *chloroplast are distributed among six genes (Table 4). The *psbA *gene contains ten introns, *rrl *and *psbC *each exhibit two introns, and *atpA*, *rrs *and *trnL(uaa) *each contains one intron. These introns vary from 309 bp to 1415 bp in size and, according to the classification system proposed by Michel and Westhof [38], fall within subgroups IA1, IA2, IA3, IB and IC3 (Table 4). Ten introns feature internal ORFs coding for putative homing endonucleases of the HNH, LAGLIDADG and GIY-YIG families (Table 4). All 17 introns, except the *rrs *intron, are located in the same positions and possess similar structures as compared to introns in other UTC algal chloroplast genomes (Fig. 3). The *rrs *intron maps to a variable region of the RNA secondary structure and to our knowledge, its insertion site has not been reported previously. The *Pseudendoclonium *chloroplast genome is the UTC algal genome the most closely related to that of *Oedogonium *in terms of intron content; these two genomes share a total of 11 introns, including eight in *psbA*.

**Table 4:** Introns in *Oedogonium *cpDNA

			**ORF**		
			
**Designation**	**Insertion site^a^**	**Subgroup^b^**	**Location^c^**	**Type^d^**	**Size (codons)**
**Group I introns**					
Oc.*atpA*.1	489	IB	L8	LAGLIDADG (2)	274
Oc.*psbA*.1	179	IA1	L9	LAGLIDADG (1)	184
Oc.*psbA*.2	276	IA1	L5	HNH	165
Oc.*psbA*.3	384	IA3	L3.2	GIY-YIG	311
Oc.*psbA*.4	408	IA1	L5	HNH	157
Oc.*psbA*.5	525	IA2	-	-	-
Oc.*psbA*.6	548	IA1	L5	HNH	202
Oc.*psbA*.7	570	IA1	-	-	-
Oc.*psbA*.8	645	IA1			
Oc.*psbA*.9	754	IA1	L5	HNH	270
Oc.*psbA*.10	898	IA1	L5	HNH	258
Oc.*psbC*.1	579	IA2	-	-	-
Oc.*psbC*.2	1009	IA2	-	-	-
Oc.*rrs*.1	426	IA2	-	-	-
Oc.*rrl*.1	1931	IA3	L8	LAGLIDADG (1)	215
Oc.*rrl*.2	2596	IA3	L6	LAGLIDADG (2)	225
Oc.*trnL*(uaa).1	35	IC3	-	-	-
**Group II introns**					
Oc.*psaC*.1	25	IIB	-	-	-
Oc.*psbB*.1	148	IIA	-	-	-
Oc.*psbI*.1	22	IIB	DIV	RT-X	458
Oc.*petD*.1	4	IIB	-	-	-

^a ^Insertion sites of introns in genes coding for tRNAs and proteins are given relative to the corresponding genes in *Mesostigma *cpDNA; insertion sites in *rrs *and *rrl *are given relative to *E. coli *16S and 23S rRNAs, respectively. For each insertion site, the position corresponding to the nucleotide immediately preceding the intron is reported.

^b ^Group I introns were classified according to Michel and Westhof [38], whereas classification of group II introns was according to Michel et al. [39].

^c ^L followed by a number refers to the loop extending the base-paired region identified by the number; D refers to a domain of group II intron secondary structure.

^d ^For the group I intron ORFs, the conserved motif in the predicted endonuclease is given, with the number of copies of the LAGLIDADG motif indicated in parentheses. For the *psbI *intron ORF, RT and X refer to the reverse transcriptase and maturase domains of group II intron-encoded proteins.

### Group II Introns

**Figure 4:**
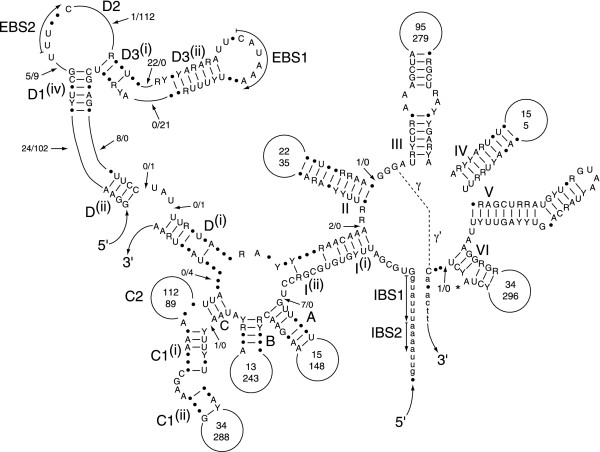
**Consensus secondary structure model of the *Oedogonium *and *Stigeoclonium psaC *introns**. The model is displayed according to Michel *et al*. [39]. Exon sequences are shown in lowercase letters. Positions exhibiting different nucleotides are denoted by dots. Positions showing deletions/additions are denoted by arrows labelled with two numbers; the left and right numbers refer to the nucleotides found in the *Oedogonium *and *Stigeoclonium *introns, respectively. Conserved base pairings are represented by dashes. Roman numerals specify the six major structural domains, whereas uppercase letters in bold denote the helices in domain I. Upper and lower numbers inside the variable loops indicate the sizes of these loops in the *Oedogonium *and *Stigeoclonium *introns, respectively. Tertiary interactions are represented by dashed lines, curved arrows, or Greek lettering. Nucleotides that potentially participate in the δ-δ' interaction are boxed. EBS and IBS are exon-binding and intron-binding sites, respectively. The putative site of lariat formation is denoted by an asterisk.

The four *Oedogonium *group II introns range from 736 bp and 2477 bp and occur in *psbB*, *psbI*, *psaC *and *petD *(Table 4). Both the putatively *trans*-spliced introns in *psaC *and *petD *feature a site of discontinuity within domain I and have positional homologues in *Stigeoclonium *cpDNA. In contrast, the presence of *cis*-spliced group II introns in the *psbB *and *psbI *gences is documented here for the first time (Fig. 3). The *psaC*, *psbI *and *petD *introns fall within subgroup IIB according to the nomenclature proposed by Michel et al. [39], whereas the *psbB *intron belongs to subgroup IIA. The predicted secondary structure of the *Oedogonium psaC *intron bears striking resemblance with that of its *Stigeoclonium *homologue; however, the *Oedogonium petD *intron shows a lower degree of similarity with the positionally homologous intron of *Stigeoclonium*. As shown by the consensus secondary structure of the *Oedogonium *and *Stigeoclonium psaC *introns (Fig. 4), the similarity observed for these introns extends well beyond the few residues expected to be conserved in members of the same subclass and their sites of discontinuity are found at the same location within domain I (between D^(*i*) ^and D^(*ii*)^). Only the *psbI *intron displays an ORF of more than 100 codons; this 458-codon ORF, located within domain IV, specifies a protein carrying a reverse transcriptase domain and a maturase domain.

### Repeated sequences

As is the case for the *Scenedesmus *chloroplast genome, that of *Oedogonium *contains fewer repeated sequences relative to its *Chlamydomonas *and *Stigeoclonium *homologues (Table 1). Repeated sequences of more than 30 bp were estimated to represent 1.28% of the *Oedogonium *genome. As in other chlorophycean genomes, these repeats map mainly to intergenic regions and most are dispersed repeats. By comparing the sequences of these repeats, we identified two repeat units (A and B). The consensus sequence of repeat unit A (ACACRTWCAATTGTAT) is identical to that of repeat unit B, except that the central region contains CATTG instead of CAATTG. Repeat unit A was identified at 82 genomic sites, whereas repeat unit B was located at 18 locations. Degenerated versions of repeat units A and B were also found in the *Oedogonium *genome. When our searches allowed a single mismatch at any position in each repeat unit, 293 and 33 copies were recovered for repeat units A and B, respectively. Although the repeats in the *Oedogonium *genome are generally isolated, some occur in tandem or are closely associated with less frequent repeat units. No repeats having identical sequences to repeat units A and B were detected in any other completely sequenced UTC algal cpDNA.

## Discussion

### The distinctive architecture of *Oedogonium *cpDNA discloses additional characters supporting the split of the Chlorophyceae into two major lineages

The architecture of *Oedogonium *cpDNA further highlights the extraordinary plasticity of the chlorophycean chloroplast genome. Compared to its *Stigeoclonium*, *Chlamydomonas *and *Scenedesmus *homologues, the 196.6-kb *Oedogonium *chloroplast genome exhibits high similarity in gene content (Table 2) but differs considerably in gene organization. Of its 99 conserved genes, only two [*trnR*(ccu) and *trnR*(ucg)], have not been found in other chlorophycean genomes. Like *Chlamydomonas *and *Scenedesmus *cpDNAs, it displays an IR; however, the patterns observed for the partitioning of genes among the two SC regions are distinctly different in these three genomes (this study, [19,21]). The *Oedogonium *chloroplast genome resembles its *Scenedesmus *homologue with reference to its tight packaging of genes and paucity of small dispersed repeats (Table 1). Among the photosynthetic UTC green algae that have been scrutinized thus far for their chloroplast genome [11,29], this oedogonialean alga shows the highest gene density and lowest proportion of dispersed repeats. Only the 71.7-kb cpDNA of the prasinophycean *Ostreococcus tauri *[40] and the 37.5-kb plastid DNA of the parasitic trebouxiophycean *Helicosporidium *sp. [41], two genomes highly reduced in gene content, have been reported to have a more compact gene organization than the *Oedogonium *genome. In contrast, the *Chlamydomonas *and *Stigeoclonium *chloroplast genomes are loosely packed and rich in small dispersed repeats. *Oedogonium *and *Stigeoclonium *cpDNAs display a plethora of introns (each containing a total of 21 introns), indicating that the degree of gene packaging has no influence on the size of intron populations observed in chlorophycean genomes. Although members of the group I intron family are predominant in both genomes, introns sharing similar structures and identical insertion positions are found at only five genomic sites (Fig. 3).

Despite its unique architecture, the *Oedogonium *chloroplast genome revealed shared derived characters (*i.e*. synapomorphies) at the levels of gene content and gene organization that reinforce our recent study based on comparative analyses of chloroplast gene sequences and gene structural features in supporting the idea that the five recognized lineages of the Chlorophyceae fall within two major clades, the CS and OCC clades [15]. The newly uncovered synapomorphic characters that provide further evidence for the close alliance of the Oedogoniales with the Chaetophorales and for their belonging to a clade separate from the CS clade are as follows: 1) the loss of *petA *from the chloroplast genome and its putative transfer to the nucleus, 2) the loss of linkage of the two distinct ORFs making up *rpoB*, 3) the gains of five derived gene clusters (one pentad, two triads and two pairs), and 4) the disruptions of two ancestral gene pairs (*rpl14-rpl5 *and *rpl2-rps19*) that are not associated with the formation of the latter derived clusters (Fig. 2). Previously, we showed that the gains of *trans*-spliced group II introns by *petD *and *psaC *and of a large insertion by *rps4 *distinguish the members of the OCC clade from those belonging to the CS clade [15]. The study reported here also revealed that the disappearance of the *psaM*, *rpl32 *and *trnL*(caa) genes in both *Chlamydomonas *and *Scenedesmus *represent additional synapomorphies that provide support for the close alliance of the Chlamydomonadales and Sphaeropleales. Furthermore, we confirmed that the duplication of *trnE*(cuu) and the acquisitions of all 11 derived clusters reported by de Cambiaire *et al*. [19], with the exception of *rps7*-*atpE*, are synapomorphies uniting the members of the CS clade. Concerning the abovementioned chloroplast genes that underwent losses, it should be pointed out that all four are uniformly present throughout the Chlorophyta, making it extremely unlikely that independent losses (*i.e*. homoplasy) rather than shared losses were responsible for the distribution patterns observed in the Chlorophyceae. The *petA *gene was reported to be missing from only *Helicosporidium*, *psaM *from *Nephroselmis*, and *trnL*(caa) from *Oltmannsiellopsis*. Given the substantial number of genomic characters that are consistent with the strongly supported conclusions derived from phylogenetic analysis of chloroplast sequence data, we conclude that there is now ample and unambiguous evidence for the split of the Chlorophyceae into two separate clades.

Nevertheless, the precise phylogenetic position of the Oedogoniales within the OCC clade remains uncertain. Phylogenomic analyses of chloroplast sequence data favoured the hypothesis that the Oedogoniales represent the first branch of the OCC clade [15]. Although this topology is supported by the finding that *trans*-spliced group II introns are present at two identical positions in the *rbcL *genes of *Stigeoclonium *and *Floydiella *but absent from their *Oedogonium *homologues, the possibility that the Chaetophorales are sister to the Oedogoniales and Chaetopeltidales could not be excluded [15]. To unravel the branching order of the lineages in the OCC clade, we are currently completing the cpDNA sequence of a representative of the Chaetopeltidales (*Floydiella*) and have undertaken the analysis of additional chlorophyceans to increase taxon sampling in phylogenomic analyses.

### The nature of the chloroplast genome in the last common ancestor of all chlorophyceans remains elusive

As observed for the IR-lacking cpDNA of *Stigeoclonium *[20], the *Oedogonium *genome proved to be too different from its *Chlamydomonas *and *Scenedesmus *counterparts and from the other previously investigated chlorophyte genomes to reconstruct with confidence the landscape of the ancestral chlorophycean genome with regard to the partitioning of genes among the SC regions, the gene order in each of these regions, and the contents in introns and small dispersed repeats. Therefore, it remains unknown whether the ancestral ulvophycean and chlorophycean genomes were similar with respect to their quadripartite structure and whether the transcription direction of the rRNA operon changed before the emergence of both the Chlorophyceae and Ulvophyceae.

The fact that the IR-containing chloroplast genomes from the representatives of the Chlamydomonadales, Sphaeropleales and Oedogoniales feature distinct gene partitioning patterns raises questions as to the factors accounting for this organizational diversity. Currently, there exists no satisfactory general model for chloroplast genome rearrangements. Given that a number of highly rearranged IR-containing cpDNAs display an increased abundance of small dispersed repeats and that the presence of small dispersed repeats and/or tRNA genes has been observed at or near the endpoints of rearranged blocks of genes, it has been proposed that these elements mediate inversions by inter- or intramolecular recombination [12,25,26,42-47]. The marked variations in sequence and abundance of small dispersed repeats in the CS and OCC lineages indicate that these repeats evolve in a very dynamic manner; however, whether they played a role in mediating the observed changes in quadripartite structure remains unknown. It is tempting to suggest that dispersed repeats were scarce in the common ancestor of all chlorophyceans and that they arose and proliferated independently in different lineages. Chloroplast genomes of closely and distantly related taxa from separate lineages will need to be investigated to understand the tempo and mode of cpDNA evolution in the Chlorophyceae. At present, only for the Chlamydomonadales do we have chloroplast genome data from more than one representative taxon [48,49]. These data, which come from physical mapping studies of a large fraction of the genes encoded by the chloroplast genome, suggest that the most phylogenetically divergent chlamydomonadalean taxa investigated (two representatives of the paraphyletic genus *Chlamydomonas*: *Chlamydomonas reinhardtii *and *Chlamydomonas moewusii*) differ by minor changes with respect to their patterns of gene partitioning between the two SC regions [50].

To our surprise, we found that the distribution of group I introns in the *Oedogonium *genome is strikingly similar to that observed for the ulvophycean *Pseudendoclonium *(Fig. 3). Whereas 11 of the 17 *Oedogonium *group I introns, including eight in *psbA*, are positionally and structurally homologous to *Pseudendoclonium *introns, only three of the *Oedogonium *introns have homologues in any of the three other completely sequenced chlorophycean cpDNAs (Fig. 3). Considering that a large fraction of the 27 group I introns found in *Pseudendoclonium *cpDNA were reported to have no known homologues when they were discovered, that they resembled one another in both secondary structure and primary sequence and that several encoded a putative homing endonuclease, intragenomic proliferation of group I introns was proposed to have occurred within the lineage leading to *Pseudendoclonium *[12]. The intron mobility conferred by the presence of homing endonuclease genes most likely explains the remarkable similarity between the group I intron contents of the *Oedogonium *and *Pseudendoclonium *genomes. Owing to this mobility, we propose that chloroplast group I introns from one chlorophyte taxon were transferred horizontally on multiple occasions to the chloroplast genome of a chlorophyte belonging to a different lineage. Because independent losses of many introns in several lineages would be expected, we consider less likely the scenario postulating that all the introns common to *Pseudendoclonium *and *Oedogonium *were present in the last common ancestor of ulvophyceans and chlorophyceans. The ability of mobile group I introns to spread readily to new genomes or new sites within a given genome together with the general property of group I introns to escape from the genomic sites they occupy through reverse splicing [51] are the main explanations for the highly variable distribution patterns observed for the genetic elements belonging to this class in chlorophyte chloroplast genomes (Fig. 3). In contrast, the rare *trans*-spliced group II introns inserted in chlorophycean genomes are stable, allowing their distribution patterns to be phylogenetically informative [15].

### The remarkably large IR of *Oedogonium *acquired non-standard chloroplast genes via horizontal DNA transfer

The increased size of the *Oedogonium *IR relative to its *Chlamydomonas *and *Scenedesmus *counterparts (Table 1) is largely accounted for by the numerous introns in *psbA *and by the internal region of about 10 kb that harbours coding sequences not normally found in the chloroplast, notably the *int *and *dpoB *genes. The predicted protein encoded by the *Oedogonium int *gene belongs to the family of tyrosine recombinases, whereas the *dpoB *product is a member of the B family of DNA-directed DNA polymerases.

Members of the tyrosine recombinase family are most widespread among bacteria and bacteriophages but also occur in archaea and eukaryotes [31,32]. The integrases of bacteriophage λ and of the conjugative transposons tn*916 *and tn*1545*, the *E. coli *resolvase XerD, and the Cre and Flp recombinases are part of this family. Tyrosine recombinases are responsible for the integration, excision or inversion of defined DNA segments [32]. To mediate site-specific recombination, these proteins recognize an inverted pair of recombination sites (20 to 30 bp in length), break and rejoin single DNA strands in pairs to form a Holliday junction intermediate. Our analyses of the *Oedogonium int *and of its immediate environment in the IR did not provide enough information to allow a judgment concerning what would be the function of the protein encoded by this gene if it is expressed. Its G+C content and codon usage deviate considerably from what we observed for the conserved protein-coding genes (Table 3), suggesting that low levels of protein, if any, would result from translation of mRNA. Furthermore, no putative recombination sites or features typical of bacterial transposons and integrons could be identified in the 10-kb region housing the unusual genes in the *Oedogonium *IR.

Genes encoding type B DNA-directed DNA polymerases are commonly found on linear mitochondrial plasmids of yeasts, filamentous fungi and higher plants. These genetic elements encode their own DNA polymerase and RNA polymerase to ensure their autonomous replication [35]. The *Oedogonium *chloroplast *dpoB *was found to be closely related to conserved protein-coding genes in terms of G+C content and codon usage (Table 3), implying that it could be actively translated if appropriate regulatory signals allow its transcription by the chloroplast RNA polymerase. The role of such translation product, however, would appear dispensable given that a chloroplast-encoded DNA polymerase dedicated to cpDNA replication is absent from all photosynthetic eukaryotes that have been examined thus far.

Because no cpDNA-encoded homologues of *int *and *dpoB *have been reported to date, the origin of these genes in *Oedogonium *is intriguing. The results of our homology searches in databases point to a mitochondrial origin. The closest homologues of the *Oedogonium int *gene was identified in the mitochondrial genomes of the green algae *Chaetosphaeridium globosum *[25] and *Prototheca wickerhamii *[33], whereas the closest homologue of the *Oedogonium dpoB *was localized on the kalilo invertron, a linear mitochondrial plasmid of *Neurospora intermedia *that can integrate into the mitochondrial genome of this fungus [36]. These observations suggest that the *Oedogonium int *and *dpoB *genes were acquired through horizontal transfer of mobile element(s) originating from the mitochondria of an unknown donor. As these genes are clustered in the same region of the *Oedogonium *IR, a single event of horizontal DNA transfer might have been responsible for their insertion in the chloroplast genome.

Although prominent insertions in the IRs of the *C. moewusii *and *Neproselmis *chloroplast genomes have also been attributed to horizontal DNA transfer events [23,52], the nature of the captured sequences remains unknown. The 21-kb insertion in the *C*. *moewusii *IR lies between the *psbA *and *rbcL *genes [53] and contains five ORFs of more than 200 codons (our unpublished results). In the *Nephroselmis *IR, a 20.9-kb region between *rbcL *and the small subunit rRNA gene (*rrl*) houses 20 ORFs of more than 80 codons, whereas a separate insertion of smaller size (6.9 kb) contains two ORFs [23]. Provided that the *Oedogonium*, *C. moewusii *and *Nephroselmis *insertions were really acquired via lateral transfer, long segments of foreign sequences would have been transferred horizontally to the chloroplast IR on at least four different occasions during the evolution of chlorophytes. As the IR often participates in intramolecular recombination events [54], such events might explain why the IR could to be a preferred target site for the insertion of foreign sequences. On the other hand, the presence of a 5.8-kb insertion carrying ORFs of unknown origin in a SC region of the *C*. *moewusii *chloroplast genome [55,56] suggests that the IR is not the only region that acquired non-standard chloroplast genes through horizontal transfer.

Our study provides the first case of horizontal gene transfer in which coding sequences of known function, not carried out by introns, were gained by the chloroplast genome in the green algal/land plant lineage. Another rare example of horizontal gene transfer involving non-standard chloroplast genes was recently reported for the cryptophyte alga *Rhodomonas salina *[57]. In this instance, a gene encoding the tau/gamma subunit of DNA polymerase III (*dnaX*) was most likely acquired from a firmicute bacterium. Only two other convincing cases of horizontal gene transfer in the chloroplast have been documented thus far; both events took place early during the evolution of algae and involved the replacement of native chloroplast genes (the *rbcL *and *rbcS *pair and *rpl36*) by bacterial genes [58,59].

## Conclusion

Our comparative analysis of the *Oedogonium *chloroplast genome with its homologues in representatives of the Chlamydomonadales, Sphaeropleales and Chaetophorales highlights the extraordinary plasticity of the chlorophycean chloroplast genome and provides compelling evidence for the dichotomy of the Chlorophyceae. No significant insight was gained into the nature of the ancestral chlorophycean chloroplast genome. Unexpectedly, our finding of two non-standard chloroplast genes within the exceptionally large *Oedogonium *IR has revealed a clear case of horizontal transfer involving most probably the capture of a mobile element from the mitochondria of a fungal or plant species. It will be interesting to see what our ongoing sequencing of *Floydiella *(Chaetopeltidales) cpDNA will tell us about the branching order of the Oedogoniales, Chaetopeltidales and Chaetophorales and about the forces shaping this genome in the OCC clade.

## Methods

### Cloning and sequencing of *Oedogonium *cpDNA

*Oedogonium cardiacum *was obtained from the Sammlung von Algenkulturen Gôttingen (SAG 575-1b) and grown in medium C [60] under 12 h light/dark cycles. An A + T rich fraction containing the cpDNA of this green alga was recovered by centrifugation of total cellular DNA in CsCl-bisbenzimide density gradients [61]. This DNA fraction was sheared by nebulization to produce 1500–2000-bp fragments that were subsequently cloned into the pSMART-HCKan plasmid (Lucigen Corporation, Middleton, WI) [24]. After hybridization of the resulting clones with the original DNA used for cloning, DNA templates from positive clones were prepared with the QIAprep 96 Miniprep kit (Qiagen Inc., Mississauga, Canada) and sequenced as described previously [62]. Sequences were edited and assembled using SEQUENCHER 4.7 (GeneCodes, Ann Arbor, MI). Genomic regions not represented in the clones analyzed were sequenced from PCR-amplified fragments. The fully annotated chloroplast genome sequence has been deposited in [GenBank:EU677193].

### Sequence analyses

Genes and ORFs were identified by Blast homology searches [63] against the non-redundant database of the National Center for Biotechnology and Information (NCBI) server. Protein-coding genes and ORFs were localized precisely using ORFFINDER at NCBI, various programs of the Wisconsin package (version 10.3) (Accelrys, San Diego, CA) and applications from the EMBOSS version 4.1.0 package [64]. Positions of genes coding for tRNAs were determined using tRNAscan-SE 1.23 [65]. Boundaries of introns were located by modelling intron secondary structures [38,39] and by comparing the sequences of intron-containing genes with those of intronless homologues using FRAMEALIGN of the Wisconsin package. For each of the four ORFs identified and for the set of 68 conserved protein-coding genes, both the codon frequency table and the base composition at the three codon positions were calculated with CUSP in EMBOSS. Codon usage bias in conserved genes and ORFs was determined using CAI [37] in EMBOSS. The values derived from the ORFs in these analyses were compared with the corresponding, average value obtained for the conserved protein-coding genes.

Forward and palindromic repeats larger than 20 bp were identified with the Comparative Repeat Analysis program [66]. Number of copies of each repeat unit was determined with FUZZNUC in EMBOSS. Populations of repeats in different chloroplast genomes were compared using the Comparative Repeat Analysis program. Regions of the genome sequence containing non-overlapping repeated elements were mapped with RepeatMasker [67] running under the WU-BLAST 2.0 search engine [68], using the repeats ≥ 30 bp identified with REPuter [69] as input sequences.

### Analyses of gene order

The sidedness index C_s _or propensity of adjacent genes to occur on the same DNA strand was determined as described by Cui *et al*. [30] using the formula C_s _= (n-n_SB_)/(n-1), where n_SB _is the number of sided blocks, *i.e*. the number of blocks including adjacent genes on the same strand of the genome, and *n *is the total number of genes. The set of genes used for this analysis did not include the *orf485*, *orf512/int*, *orf538/dpoB *and *orf1252*. Conserved gene pairs or gene clusters exhibiting identical gene polarities in selected green algal cpDNAs were identified using a custom-built program.

## Authors' contributions

J–SB participated in the conception of this study, performed most of the sequence analyses, generated the tables and figures and drafted the manuscript. CO performed the sequencing and contributed to the assembly and annotation of the genome sequence. CL and MT conceived and supervised the study, contributed to the analysis and interpretation of the data and prepared the manuscript. All authors read and approved the final manuscript.
